# *The tps5*, *tps10* and *tps11* class II trehalose phosphate synthase mutants alter carbon allocation to starch and organic and amino acids at two different photoperiods in *Arabidopsis*

**DOI:** 10.1007/s00425-025-04705-1

**Published:** 2025-05-02

**Authors:** Andrea C. Ruiz-Castillo, Daniela J. Bonilla-Córdoba, Ismael Cisneros-Hernández, Norma Martínez-Gallardo, Enrique Ramírez-Chávez, John Délano-Frier

**Affiliations:** Centro de Investigación y de Estudios Avanzados del IPN, Unidad Irapuato; Km 9.6 del Libramiento Norte Carretera Irapuato-León, C.P. 36821 Irapuato, Gto México

**Keywords:** Class II trehalose phosphate synthase genes, Carbon and nitrogen allocation, Nitrate reductase, Non-structural carbohydrates, Organic and amino acids, Short- and long day photoperiods

## Abstract

**Main conclusion:**

Altered C and N allocation in response to short- and long photoperiods in class II TPS mutants suggest that they negatively regulate the TPS1-Tre6P metabolic regulator system in *A. thaliana*.

**Abstract:**

The biological function of class II TPS genes remains largely enigmatic, although there is evidence that they may play an important regulatory role in plant stress responses as well as in development and growth. Recent findings indicated that part of biological function of TPSII proteins may be related to their capacity to associate with the SnRK1 regulator of metabolism in order to inhibit its nuclear activity. The results of the present study show that insertional mutants of the *TPS5*, *TPS10* and *TPS11* class II *TPS* genes had a marked effect on the carbon allocation to non-structural carbohydrates, notably starch, and to organic and amino acids during both short- and long-day photoperiods. The results obtained in this study, which resembled those obtained previously in *AhTPS1* overexpressing plants, suggest that these particular TPSII proteins may negatively regulate of C and N allocation to non-structural carbohydrates, organic and amino acids mediated by the TPS1-Tre6P central metabolic regulator system in *A. thaliana* plants. The effect observed was sometimes dependent on of the photoperiod employed and the mutant examined. The mechanism by means of which these TPS II proteins may specifically target TPSI activity and Tre6P levels in order to regulate C and N allocation in *A. thaliana* in response to short- and long-day photoperiods remains to be determined.

**Supplementary Information:**

The online version contains supplementary material available at 10.1007/s00425-025-04705-1.

## Introduction

Trehalose-6-phosphate (Tre6P) is a central signaling molecule that responds to sucrose availability in order to regulate carbon (C) metabolism mostly by controlling sucrose (Suc) utilization and allocation and, therefore, plant growth and reproductive success (Schluepmann et al. 2004; Figueroa and Lunn 2016; Paul et al. 2020, 2022). Solid experimental evidence suggests that Tre6P positively regulates growth by inhibiting the influence of sucrose-non-fermenting1-related kinase1 (SnRK1), a master metabolic regulator that promotes catabolic processes, while concurrently repressing anabolic metabolism, in response to low energy conditions. Thus, by blocking SnRK1, Tre6P is capable of positively promoting growth and development (Zhang et al. 2009; Lawlor and Paul 2014; Morales-Herrera et al. 2023). While there are 11 *TPS* genes in *Arabidopsis thaliana* (i.e., *AtTPS1*-*AtTPS11*), only a number of those included in class I *TPS*, i.e., *AtTPS1* to *AtTPS4*, are known to code for trehalose-6-phosphate synthases (TPS) capable of synthesizing T6P from UDP-glucose and Glc-6-phosphate (Leyman et al. 2001; Fichtner and Lunn 2021, and references therein). Their importance is highlighted by the severe phenotypes produced in mutants having reduced or null AtTPS1 activity, which range from dwarfish growth and delayed development to anomalous cell wall morphology, embryo lethality and delayed flowering (Eastmond et al. 2002; van Dijken et al. 2004; Gómez et al. 2006, 2010; Wahl et al. 2013). In contrast, no convincing evidence has been produced to show that class II TPS proteins have TPS activity. Class II TPS proteins in *Arabidopsis* and other flowering plants are divided into at least two separate clades, with AtTPS5-AtTPS7 clustering separately from AtTPS8-AtTPS10, while the phylogenetic relationship of AtTPS11 remains undecided (Yang et al. 2012; Henry et al. 2014; Liu et al. 2019). Apart from a handful of reports suggesting possible mechanisms of action, the biological function of class II *TPS* genes remains largely enigmatic. However, the confirmed transcriptional regulation of the class II *TPS* genes, characterized by tissue-specific expression and responsiveness to carbon availability and phytohormones is clearly indicative of their important regulatory role in plant development and growth. Several studies have provided solid experimental evidence to support this proposal. Thus, *AtTPS5* was shown to participate in basal defense regulation of *A. thaliana* defenses against necrotrophic and biotrophic pathogens (Wang et al. 2019), in the negative regulation of abscisic acid (ABA) signaling leading to altered trehalose content and nitrate reductase activity (Tian et al. 2019), and in thermotolerance, involving trehalose-, salicylic acid-, and ethylene-related signaling pathways (Suzuki et al. 2008). *AtTPS6* was shown to play a role in plant development and to regulate cellular morphogenesis and plant architecture (Chary et al. 2008), *OsTPS8* improved salt tolerance in rice, by increasing suberin deposition and the expression of ABA-responsive genes (Vishal et al. 2019), while the over expression of *TPS8* in *Brassica napus* increased low N and high Suc-induced anthocyanin accumulation (Yuan et al. 2024a) and enhanced biomass accumulation and seed yield with an augmented seed oil content by directing the partition of assimilated carbon into starch and Suc (Yuan et al. 2024b). Finally, a *TPS11* gene from a freezing-tolerant wheat cultivar modified carbohydrate metabolism and conferred increased protection to cold temperatures when overexpressed in *A. thaliana* (Liu et al. 2019), whereas aphid-resistance against green peach aphids in *A. thaliana* was dependent on a trehalose-based defense response regulated by *AtTPS11* in conjunction with the *PHYTOALEXIN DEFICIENT4* defense-modulating gene that favored the allocation of carbon into starch rather than Suc (Singh et al. 2011). Moreover, class II *TPS* genes in sweet orange produced varying expression patterns in response to phytohormones and/or various stress conditions (Liu and Zhou 2022), whereas the expression levels of four *TPSII* genes in *B. napus* increased sharply after drought stress, while other two showed variable expression patterns among source and sink tissues (Yang et al. 2023). Finally, TPSII proteins were found to strongly associate with the SnRK1 complex in order to inhibit its nuclear activity by various possible mechanisms (Van Leene et al. 2022).

In this study, *A. thaliana tpsII* mutants, i.e., *tps5-1*, *tps10-2* and *tps11-2* belonging to the three main subgroups in which this gene family is generally thought to be divided, were exposed to short day (8 h light/ 16 h dark) and long day (16 h light/8 h dark) photoperiods to determine their effect on carbon and nitrogen metabolism in terms of their allocation into soluble and insoluble nonstructural carbohydrates, organic acids and amino acids, in addition to their effect on the activity of nitrate reductase enzyme and diverse photosynthetic parameters. The results exposed several differences in the patterns of accumulation/depletion of these primary metabolites between WT control plants and the *tpsII* mutants and sometimes between the individual mutants as well. They also indicated that *TPSII* genes play an important role in the regulation of C and N metabolism in plants in response to short- and long-day photoperiods. The mechanisms by means of which this metabolic regulation might be taking place will require further experimentation to be determined with greater clarity.

## Material and methods

### Plant material and growth conditions

The insertional mutants of the *A. thaliana TPSII* genes were provided by the mutant bank of the *Arabidopsis* Biological Resource Center (ABRC). The insertional mutant lines were part of the SALK (Alonso et al. 2003), GABI-KAT (Rosso et al. 2003), and *WiscDsLox* (Woody et al. 2007) collections. Two insertional lines of each *tpsII* mutant were provided, which were selected based on the primary criterion of having the insertion localized within the codifying region of the gene and positioned as close as possible to the start codon. Homozygous lines of each mutant were generated from 25 seeds of the original lines, using specific oligonucleotides for genotyping purposes (Table S1). Only the progeny of those plants, termed *tps5-1*, *tps10-2* and *tps11-2*, that confirmed to be homozygous for the insertion allele of the *tpsII* mutants were selected for further experimentation (Fig. S1). Another criterion employed for their selection was based on preliminary results showing the highest sensitivity to changes non-structural (NSC) carbohydrate levels in response to changing photoperiods.

Seeds of wild-type (WT) and *tpsII* mutant *A. thaliana* plants were germinated in 100 × 15 mm Petri dishes containing 0.1 × MS media (Murashige and Skoog 1962), pH 5.7, prepared with 0.43 g/l of MS salts, 0.5% de Suc and 1% agar. The Petri dishes were kept in a conditioned growth room maintained at 22 ± 1ºC, with a 16 h light/ 8 h dark basal photoperiod. Illumination was provided by white light fluorescent luminaries (ECOFIT T8; Philips; Amsterdam, The Netherlands) having a fluence rate of 29.3 µmol m^−2^ seg^−1^. Seedlings were cultivated under these conditions for ten days post-germination (dpg), and were then transferred to 125 ml plastic pots containing a substrate prepared with “Sunshine Mix 3” (SunGro Horticulture, Bellevue, WA, USA), silt, mulch, vermiculite [SunGro Hort] and perlite (Termolita S.A., Nuevo León, México) in a 3/1/2/1/1 (by vol.) proportion and were fertilized with 20:10:20 (N:P:K) water soluble fertilizer according to the manufacturer´s instructions (Peters Professional; Scotts-Sierra Horticultural Products, Marysville, OH, USA). Sixty g of this substrate was added to each pot to ensure the uniform growth of the plants. Immediately after three seedlings were transferred to the plastic pots, they were returned to the conditioned growth room for a 10-day adaptation period under the standard growing conditions (i.e., 16 h light/8 h dark, at 22 °C). At the end of this period groups of plants were transferred to the experimental stage, performed in a home-made growth chamber maintained at 22 °C and programmed to operate under two different photoperiods: 16 h light and 8 h dark = “long day”, and 8 h light and 16 h dark = “short day”. Groups of 90 plants of each genotype were used for each photoperiod examined. Each experiment was repeated at least twice. Thirty rosettes were sampled at each of the three sampling time-points included in the short- and long-day photoperiods, respectively. Harvested rosettes were flash frozen in liquid nitrogen, ground to a fine powder in a frozen mortar and pestle and stored at -70°C until further analysis. Prior to freezing, sampled rosettes were divided into two pools consisting of 15-plant replicates each, which were analyzed as such in each time-point examined per photoperiod.

Rosette areas of WT and *tpsII* mutant plants were measured 25 days post-germination (dpg) under long-day conditions at 22 ± 1ºC. Photographs of each individual rosette were used to calculate their areas, which was performed using the ImageJ software (Rasband, W.S., ImageJ, U.S. National Institutes of Health, Bethesda, Maryland, USA, https://imagej.net/ij/, 1997–2018). Flowering time was determined by measuring the number of flower buds that were generated after the initiation of the flowering period, which occurred 26 dpg. A total of fifty plants were measured for each genotype in the vegetative and reproductive development experiments, which were repeated thrice.

### Metabolite extraction and measurement

#### Non-structural carbohydrates (NSCs)

NSCs were analyzed according to enzymatic-coupled method as described previously (Cisneros-Hernández et al. 2021). Briefly, soluble NSCs were analyzed from extracts produced from 20 mg of lyophilized ground tissue extracted thrice with 600 µl of a 50 mM Hepes KOH buffer, pH 7.4, containing 5 mM MgCl_2_ in 80% aqueous ethanol (v/v). After extraction and clarification by centrifugation, the resulting supernatants were combined. Sucrose, glucose (Glc), and fructose were determined enzymatically in the supernatants using a coupled assay with glucose-6-phosphate dehydrogenase (from yeast, grade II, Roche, Mannheim, Germany) in which NADPH formation was measured at 340 nm (Zhao et al. 2010). For starch, the pellets resulting from the above procedure were resuspended in 0.5 ml of 10 mM KOH and autoclaved for 30 min. Starch was hydrolyzed overnight at 37 °C in a starch degradation buffer consisting of 100 mM Hepes, pH 7.5, 3 mM MgCl_2_, 10 U of α-amylase, Type VI-B, from porcine pancreas (E.C. number 3.2.1.1; Sigma-Aldrich Chemical, St. Louis, MO, USA) and 10 U of amyloglucosidase from *Aspergillus niger* (E.C. number 3.2.1.3; Sigma-Aldrich). After centrifugation, the supernatants were stored at 4 °C. The pellet was extracted again with 0.5 ml of starch degradation buffer and incubated at 37 °C for 30 min. Following centrifugation, both supernatants were combined and assayed enzymatically for Glc as described above.

### Organic acids and amino acids

Fifty mg of pulverized frozen plant tissue were placed in previously chilled 1.5 ml Eppendorf tubes and covered with 500 µl of 80% aqueous ethanol (v/v) plus 20 µl of an internal standard solution, i.e., norleucine, at 0.1 mg/ml, dissolved in the same ethanolic solution. Subsequently, all samples were sonicated for 30 min followed by an extraction at 80 °C for 30 min in a thermo-block (Eppendorf ThermoMixer F2.0, Hamburg, Germany), a subsequent cooling period at room temperature and centrifugation at 9.6 × g for 7 min. The clear supernatants were recovered and were dried under vacuum in a Maxi-Dry Lyo centrifuge (Heto-Holten, Allerød, Denmark) during 6 h. Twenty µl of pyridine were added to the dry samples in addition to 100 µl of N,O-bis(trimethylsilyl)trifluoroacetamide and were incubated at 80 °C together with agitation at 550 rpm for 30 min in a thermo-block. Past this time, the extracts were transferred to 300 µl glass vials, posterior to the addition of 100 µl of iso-octane, and were then analyzed by GC–MS. This was performed using a gas chromatograph (Agilent Technologies model 7890A; Santa Clara, CA, USA), coupled to a mass spectrometer (Hewlett Packard, model 5975C; Palo Alto, CA, USA), equipped with a 60 m length × 250 µm de diameter × 0.25 µm of film thickness J and W DB-1 ms non-polar 100% dimethylpolysiloxane column (Agilent Technologies). Chromatography-grade helium gas was used as the mobile phase.

All GC–MS data were analyzed by the MSD ChemStation F.01.01.2317 software to generate a "Total Ion Chromatogram" (TIC), showing the total of ions generated per time at a rate of 3 scans/s. The TICs generated were then analyzed by *Automated Mass Spectral Deconvolution and Identification System,* o *AMDIS*, program which separated the peaks detected in order to group the ions in function of their binomial distribution, allowing for the “noise” ions to be discarded. The grouped ions were then converted into their respective mass spectra in order to compare them to the information contained in the *Mass Spectral Database (NIST v11,* “National Institute of Standards and Technology”, Gaithersburg, MD, USA). The spectra were classified in descending orders of similitude to identify the best match of the compounds present in accordance with their retention time.

### Nitrate reductase (NR) assay

NR activity was measured according to a method developed by Kim and Seo (2016) that is based on the colorimetric determination of nitrite, its final product. Thus, nitrite concentration is measured by its absorbance at 540 nm, formed by a series of reactions involving the initial formation of a diazonium salt by the reaction of nitrite with sulfanilic acid, that subsequently produced a colored azo compound in the presence of N-(1-naphthyl) ethylenediamine dihydrochloride.

### Determination of photosynthetic parameters

The gas exchange measurements were conducted between 9:00–11:00 a.m. (central local time in México) using an infrared gas analyzer-coupled portable CIRAS-3 photosynthesis system (PP Systems, Amesbury, MA, USA). The intracellular CO_2_ concentration (Ci), stomatal conductance (gs), transpiration rate (E), water use efficiency (WUE), vapor pressure deficit (VPD), and net photosynthetic rate (A) under saturated irradiance were recorded under a photosynthetic photon flux density of 1000 μmol m^−2^ s^−1^. Other conditions were as follows: ambient CO_2_ concentration, 380 μmol/mol; air temperature, 25 °C; cuvette air flow rate, 500 ml min^−1^, and a 55% relative air humidity.

### Statistical analysis

All experiments were performed utilizing a completely randomized design, in which the 30 or 50 plants required for each experimental stage were taken from a total population of 120 plants per genotype that were routinely propagated in an ambient-controlled growth room. All data was subjected to ANOVAs, followed by Tukey HSD tests (*P* ≤ 0.05). The Bonferroni correction (Bonferroni 1934) was employed to adjust the probability (*P*) values when making multiple statistical tests. The R (http://r-project.org/) and Rstudio (https://www.rstudio.com) statistical programs were used.

## Results

### Modified vegetative and reproductive development in the *tpsII* mutants

**Fig. 1:**
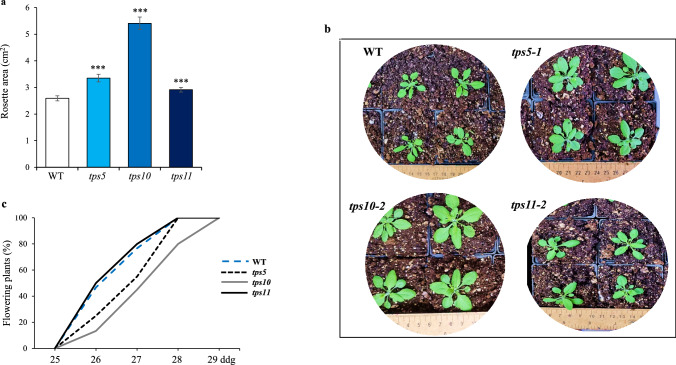
Vegetative and reproductive development in *tpsII* gene mutants in *A. thaliana* **a**,** b** Rosette diameters of the *tps5-1*, *tps10-2* y *tps11-2* insertional mutants and wild-type (WT) controls as measured using Image J software. **c** Flowering time, measured 25 days after germination in the *tpsII* mutants and *WT* plants. Fifty plants per genotype were used for each measurement. The bars shown in **b** represent the average ± SE (*n* = 50). Asterisks over the bars denote significant differences (one-way ANOVA and Tukey test, *P* ≤ 0.05 [*], *P* ≤ 0.01 [**] or *P* ≤ 0.001 [***]). The experiment was repeated at least twice using 50 plants per genotype in each replica, all of which yielded similar results

The three *tpsII* mutants showed significantly greater vegetative growth than control WT plants (Figs. 1a and b). The effect was more pronounced in the *tps10-2* mutant, whose rosette area was approximately 2 times larger than WT plants. Increased vegetative growth in this mutant was inversely related to flowering time, which was clearly retarded with respect to all other genotypes examined, reaching 100% flowering with an approximately 2 days delay (Fig. 1c). The start of the flowering transition in the *tps5-1* mutant was also slow, but contrary to the *tps10-2* mutants, it was able to recover sooner.

### Variations in soluble and insoluble non-structural carbohydrate (NSC) content in the *tpsII* mutants during short- and long-day photoperiods

**Fig. 2:**
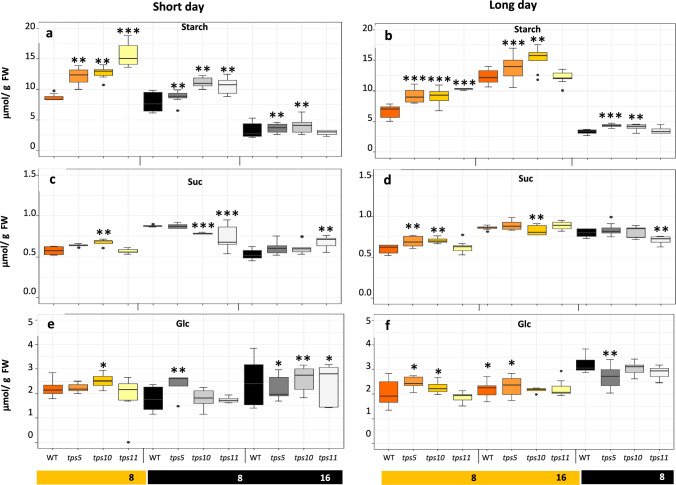
Soluble and insoluble non-structural carbohydrates accumulation in *A. thaliana tpsII* gene mutants during short- and long day-photoperiods Fluctuations in starch (**a**, **b**), sucrose (Suc; **c**, **d**) and glucose (Glc; **e**, **f**) contents were measured during the duration of short- (**a**, **c**, **e**) and long-day (**b**, **d**, **f**) photoperiods in leaves of the *tps5-1*, *tps10-2* and *tps11-2* insertional mutants and in WT controls. Box-and-whisker plots show high, low, and median values (*n* = 30). Asterisks over the box- and-whisker plots denote significant differences (one-way ANOVA and Tukey test, *P* ≤ 0.05 [*], *P* ≤ 0.01 [**] or *P* ≤ 0.001 [***]). Each biological replica of the experiment was performed at least twice using 90 plants per genotype, 30 of which were collected in each of the three time points selected for analysis in the short and long-day photoperiods. Each group of 30 plants was further divided into two pools of 15 plants for analysis. All experimental replicas yielded similar results

Leaf starch content was the NSC whose content was more readily modified in all the *tpsII* mutants examined, with a constant tendency to significantly accumulate above WT starch levels during the exposure to light. independently of the photoperiod used (Figs. 2a and 2b). The single exception was detected in of the *tps11-2* mutant at the end of the long day, whose starch levels were no different than WT plants (Fig. 2b). Starch levels in these *tpsII* mutants remained mostly higher than WT plants after the transition to the short and long night periods (Figs. 2a and b). The content of soluble NSCs in the *tpsII* mutants with respect to WT did not vary as clearly and constantly as starch. The most constant effect observed was the significant increase in Suc levels detected in the *tps10-2* mutant in the short light periods examined and the minor effect that the exposure to the dark had on Suc contents, which did not vary much from those detected during the short- and long-days (Figs. 2c and 2d). A similar pattern was observed regarding Glc levels that, in general, significantly accumulated above WT levels during the short- and long-day periods in the *tps5-1* and *tps10-2* mutants but remained unchanged in the *tps11-2* mutant. Similar to Suc, Glc levels were, in general, not reduced during the night and reached significantly higher contents than WT plants in the *tps10-2* and *tps11-2* mutants at the end of the long-night, contrary to the *tps5-1*, whose Glc levels were significantly lower than WT (Figs. 2e and 2f).

### Variations in organic acids and amino acids content in the *tpsII* mutants during short- and long-day photoperiods

#### Tricarboxylic acid (TCA) pathway organic acids and glyceric acid

**Fig. 3:**
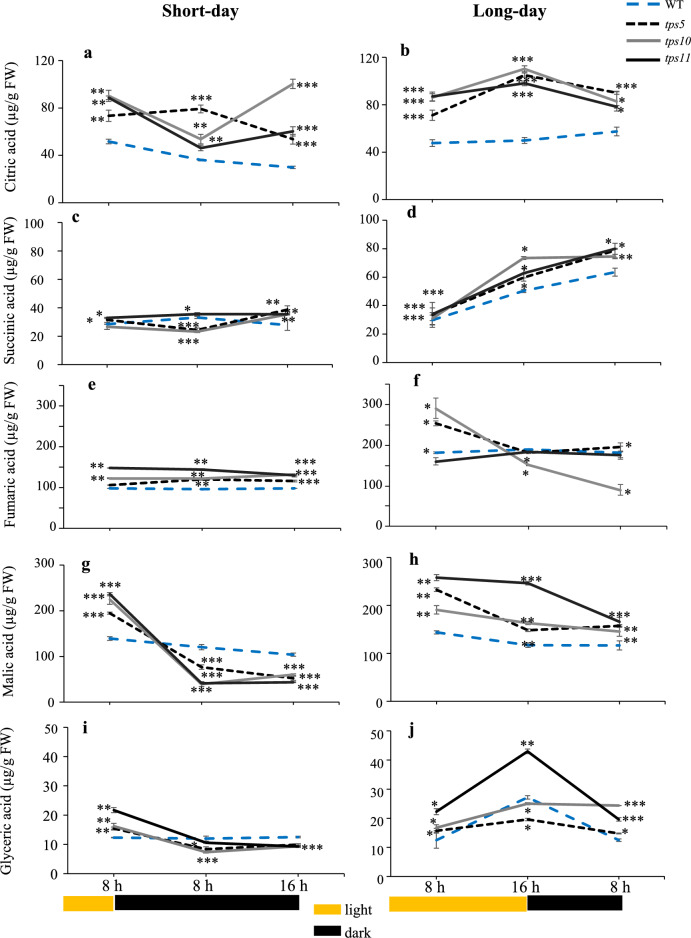
Temporal levels of tricarboxylic acid (TCA) pathway-associated organic acids and glyceric acid in *A. thaliana tpsII* gene mutants during short- and long day-photoperiods. Extracts from the *tps5-1*, *tps10-2* y *tps11-2* insertional mutants and from WT controls were prepared from leaves sampled every 8 h for 24 h during short- and long-day photoperiods. Citric acid (**a**, **b**), succinic acid (**c**, **d**), fumaric acid (**e**, **f**), malic acid (**g**, **h**), and **i**glyceric acid (**i**, **j**) were determined using gas chromatography, linked to tandem mass spectrometry. Values are presented as means ± SE (*n* = 30). Asterisks denote significant differences (one-way ANOVA and Tukey test, *P* ≤ 0.05 [*], *P* ≤ 0.01 [**] or *P* ≤ 0.001 [***]). The Bonferroni correction was applied to adjust the significance level and to control the influence of type I error in multiple comparisons. Statistics as in Fig. 2

The content of citrate, succinate, fumarate and malate in WT controls remained mostly constant in the short- and long-day photoperiods, whereas succinic acid gradually accumulated to reach highest levels at the end of the night of the long-day (Figs. 3a to 3d). Barring a few exceptions, i.e., the fall in fumarate in the *tps10-2* mutant at the end of the long day and short night (Fig. 3f), respectively, the reduction of succinate in the *tps5-1* and *tps10-2* at the end of the first 8 h of the long night (Fig. 3d), and the drastic fall of malate during the long night in all three *tpsII* mutants (Fig. 3g), most TCA-related organic acids examined accumulated to significantly higher levels in the *tpsII* mutants than in the WT controls during the extension of the two photoperiods examined. This trend prevailed during the night periods, when their levels mostly remained constant or increased, except for the negative effect that the long night had on malate levels in the three *tpsII* mutants, as mentioned above. Glyceric acid levels also showed a marked tendency to be significantly reduced during the long night in all three *tpsII* mutants tested (Fig. 3i) and, similarly to malate, the three *tpsII* mutants had significantly higher levels of glycerate than WT controls at the end of the short-night (Fig. 3j). Also relevant was the peak of malate and glycerate detected in the *tps11-2* mutant at the end of the long day and the fact that glyceric acid contents in the *tps5-1* mutant remained practically unchanged throughout the two photoperiods. although in several occasions it significantly dipped below those detected in WT controls (Figs. 3i and 3j).

### Amino acids derived from pyruvic acid and the malate/ oxalacetate valve

**Fig. 4:**
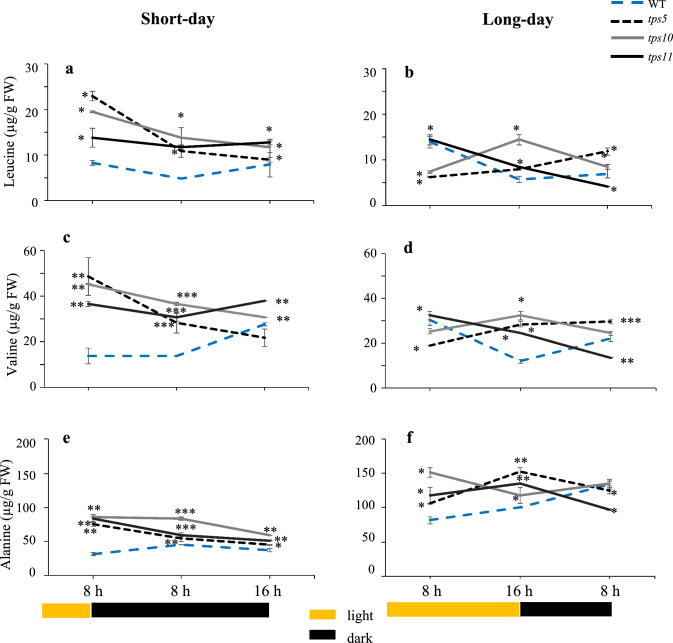
Temporal levels of pyruvate-derived leucine, valine and alanine amino acids in *A. thaliana tpsII* gene mutants during short- and long day-photoperiods Extracts were from the *tps5-1*, *tps10-2* y *tps11-2* insertional mutants and from WT controls were prepared from leaves sampled every 8 h for 24 h during short- and long-day photoperiods. Leucine (**a**, **b**), valine (**c**, **d**) and alanine (**e**, **f**) were determined using gas chromatography, linked to tandem mass spectrometry. Values are presented as means ± SE (*n* = 30). Asterisks denote significant differences (one-way ANOVA and Tukey test, *P* ≤ 0.05 [*], *P* ≤ 0.01 [**] or *P* ≤ 0.001 [***]). The Bonferroni correction was applied to adjust the significance level and to control the influence of type I error in multiple comparisons. Statistics as in Fig. 2

In WT plants, the content of the pyruvate-derived amino acids leucine (Leu) and valine (Val) remained relatively stable in the two photoperiods examined (Figs. 4a to 4d). Alanine (Ala) levels in WT changed very little during the short- and long-days and showed a tendency to increase during the night periods (Figs. 4e and 4f). Most *tpsII* mutants accumulated significantly higher levels of Leu, Val and Ala during the short- and long-days, except for the slightly significant reduction, with respect to WT controls, of Ala in the *tps5-1* mutant and of the three amino acids in the *tps11-2* mutant at the end of the short night (Figs. 4b, 4d and 4f).

**Fig. 5:**
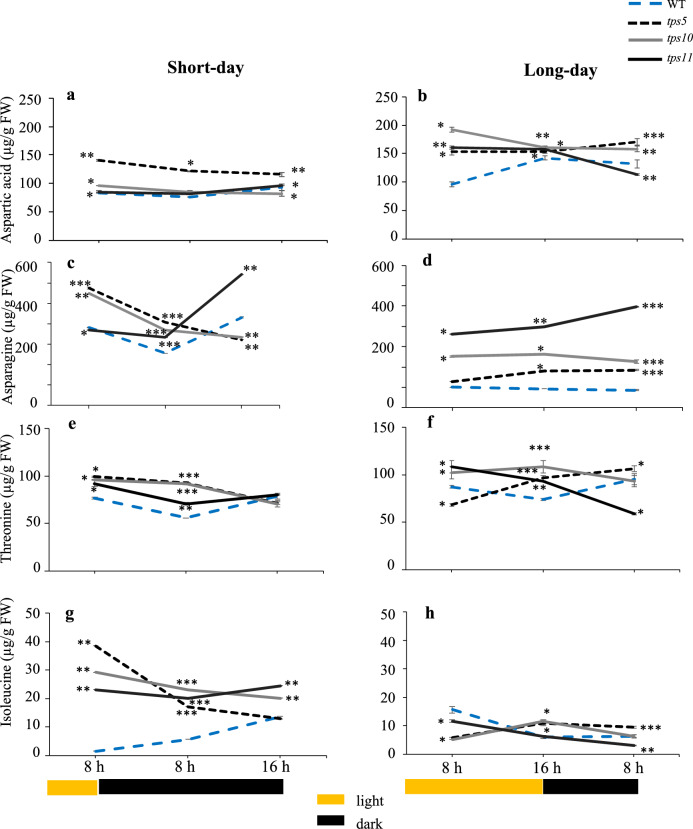
Temporal levels of pyruvate-derived aspartic acid, asparagine, threonine and isoleucine alanine amino acids in *A. thaliana tpsII* gene mutants during short- and long day-photoperiods extracts from the *tps5-1*, *tps10-2* y *tps11-2* insertional mutants and from WT controls were prepared from leaves sampled every 8 h for 24 h during short- and long-day photoperiods. Aspartic acid (**a**, **b**), asparagine (**c**, **d**), threonine (**e**, **f**) and isoleucine (**g**, **h**), were determined using gas chromatography, linked to tandem mass spectrometry. Values are presented as means ± SE of two independent biological replicates (*n* = 30). Asterisks denote significant differences (one-way ANOVA and Tukey test, *P* ≤ 0.05 [*], *P* ≤ 0.01 [**] or *P* ≤ 0.001 [***]). The Bonferroni correction was applied to adjust the significance level and to control the influence of type I error in multiple comparisons. Statistics as in Fig. 2

Aspartate (Asp) levels in WT controls reached highest levels at the end of the long day in the long-day photoperiod, whereas they remained constant during the short-day photoperiod (Figs. 5a and 5b). In contrast, WT asparagine (Asn) levels remained constant during the long-day photoperiod but were transiently reduced at the end of the first 8 h of the night stages of the short-day photoperiod (Figs. 5c and d). In general, Asp levels in the *tpsII* mutants were higher than those in WT controls and tended to reach the lowest levels at the end of the night of both the short- and long-day photoperiods; these were significantly lower than WT in the *tps10-2* and *tps11-2* mutants at the end of the long and short nights, respectively. Asn changes in the *tpsII* mutants were more drastic and were clearly influenced by the photoperiod. Thus, in the short-day, a clear tendency to reduce their Asn levels in the night period was evident in the *tps5-1* and *tps10-2* mutants, which became significantly lower than WT at the end of the long night, contrary to the *tps11-2* mutant that reached its highest Asn levels at this same time-point. Conversely, in the long-day photoperiod, Asn levels were consistently and significantly higher than WT controls and, again, reached their highest levels at the end of the short night in the *tps11-2* mutant.

Threonine (Thr) levels were relatively constant in WT plants, while they maintained significantly higher levels at the end of the short day and its subsequent 8 h dark stage in the three *tpsII* mutants. In general, significantly highest levels of Thr were also detected throughout the duration of the long day photoperiod, except for the significantly reductions detected in the *tps5-1* and *tps11-2* and mutants at the end of the first 8 h of the long day and at the end of the short night, respectively (Figs. 5e and 5f). Isoleucine (Ile) accumulation patterns were similar, with WT control levels remaining more or less constant irrespective of the type and phase of the photoperiod. However, Ile content in the two photoperiods was drastically different in the *tpsII* mutants: in the short-day photoperiod, they were clearly and significantly higher than WT controls despite the tendency to gradually decrease as the photoperiod progressed (Fig. 5g), while no great differences in Ile contents were recorded between WT and the *tpsII* mutants during the long day, although the tendency to decrease in the dark remained (Fig. 5h).

### Amino acids derived from the photorespiratory pathway and/ or involved in nitrogen fixation

**Fig. 6:**
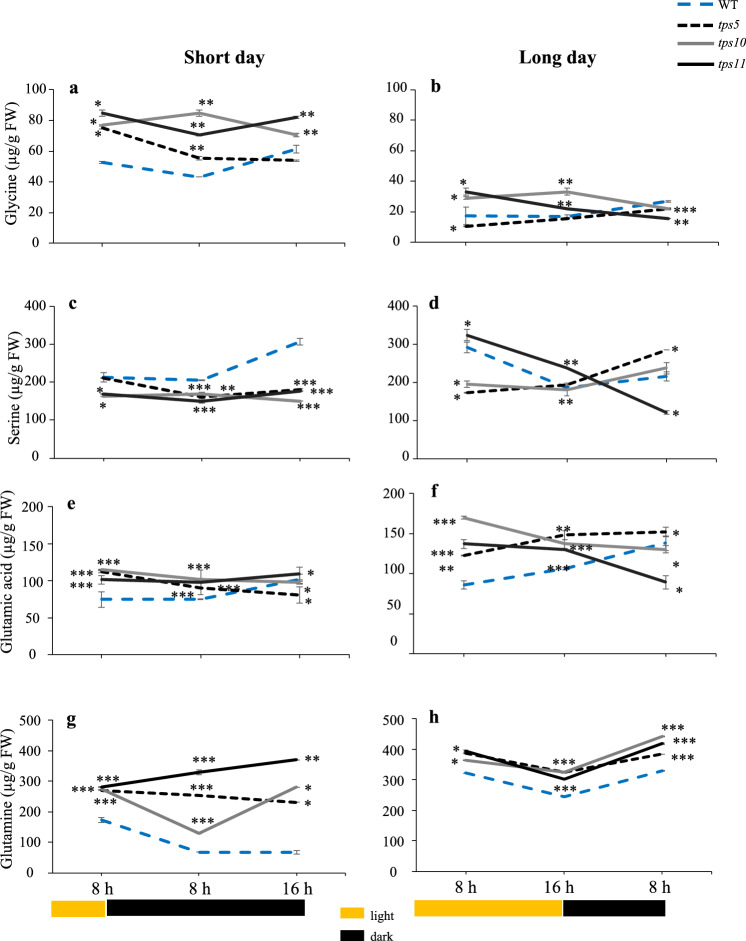
Temporal levels of photorespiration-associated glycine and serine, and of nitrogen-fixation associated glutamic acid and glutamine amino acids in *A. thaliana tpsII* gene mutants during short- and long day-photoperiods Extracts were from the *tps5-1*, *tps10-2* y *tps11-2* insertional mutants and from WT controls were prepared from leaves sampled every 8 h for 24 h during short- and long-day photoperiods. Glycine (**a**, **b**), serine (**c**, **d**), glutamic acid (**e**, **f**) and glutamine (**g**, **h**) were determined using gas chromatography, linked to tandem mass spectrometry. Values are presented as means ± SE of two independent biological replicates (*n* = 30). Asterisks denote significant differences (one-way ANOVA and Tukey test, *P* ≤ 0.05 [*], *P* ≤ 0.01 [**] or *P* ≤ 0.001 [***]). The Bonferroni correction was applied to adjust the significance level and to control the influence of type I error in multiple comparisons. Statistics as in Fig. 2

Glycine (Gly) levels in WT plants tended to increase as the dark periods extended. This was contrary to the pattern of accumulation observed on the *tpsII* mutants which, in general, tended to reach lowest Gly levels at the end of the night periods (Figs. 6a and 6b). This was particularly evident at the end of the long-day night, where all *tpsII* mutants reached significantly lower Gly levels than WT controls. The same tendency was observed in the short-day photoperiod, although in this case none of Gly levels detected in the mutants was significantly lower than that in WT controls. In WT plants, serine (Ser) levels decreased or remained constant during the day of both photoperiods and gradually increased during the night (Figs. 6c and 6d). Ser levels in the short day were predominantly lower than WT controls in all *tpsII* mutants throughout the whole photoperiod, with a tendency to increase in the night, except in the *tps10-2* mutant. However, in the long day, Ser contents in the *tps5-1* and *tps10-2* mutants, although in general significantly lower than in WT controls during the day, gradually increased to reach highest levels at the end of the short night, which were significantly higher than WT controls in the *tps5-1* mutant. The *tps11-2* mutants had a completely contrasting behavior, having their highest and lowest Ser levels at the start and end of the long day, respectively.

Glutamate (Glu) levels in WT plants gradually increased in both photoperiods and reached their highest levels at the end of their respective nights. In general, all *tpsII* mutants accumulated significantly higher Glu levels than WT controls, mostly during the light periods. However, except for the *tps5-1* mutant during long-day photoperiod, they all showed a tendency to reduce their Glu contents as the photoperiods progressed (Figs. 6e and 6f). Glutamine (Gln) contents were also significantly higher than WT controls in all three *tpsII* mutants in both photoperiods tested, but contrary to Glu, they predominantly reached their highest levels at the end of the short and long nights, respectively (Figs. 6g and 6h).

### Variations in nitrate reductase (NR) activity during short- and long-day photoperiods

**Fig. 7:**
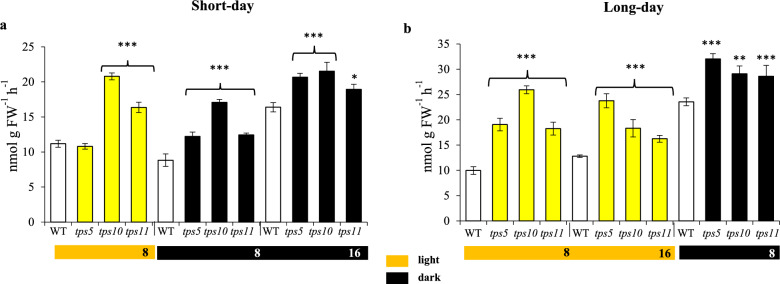
Nitrate reductase (NR) activity levels in *A. thaliana tpsII* gene mutants during short- and long day-photoperiods NR activity, measured in leaf extracts from plants grown under short- (**a**) and long-day (**b**) photoperiods in the *tps5-1*, *tps10-2* y *tps11-2* insertional mutants and WT controls. The bars represent the average ± SE (*n* = 30). Asterisks over the bars denote significant differences (one-way ANOVA and Tukey test, *P* ≤ 0.05 [*], *P* ≤ 0.01 [**] or *P* ≤ 0.001 [***]). Statistics as in Fig. 2

NR activity is ubiquitous in plants, and is involved in the crucial conversion of nitrate to ammonia, which will eventually be incorporated into Glu and other carbon-skeleton acceptors. In general, NR activity remained relatively stable in WT plants, although it showed a tendency to slightly increase its activity levels during the dark. In contrast, significant higher NR activity levels were detected in the three *tpsII* mutants analyzed during the course of the short- and long-day photoperiods. Highest NR activities in the *tpsII* mutants were predominantly reached at the end of the short and long nights, respectively (Fig. 7).

### Variations in photosynthetic parameters

**Fig. 8:**
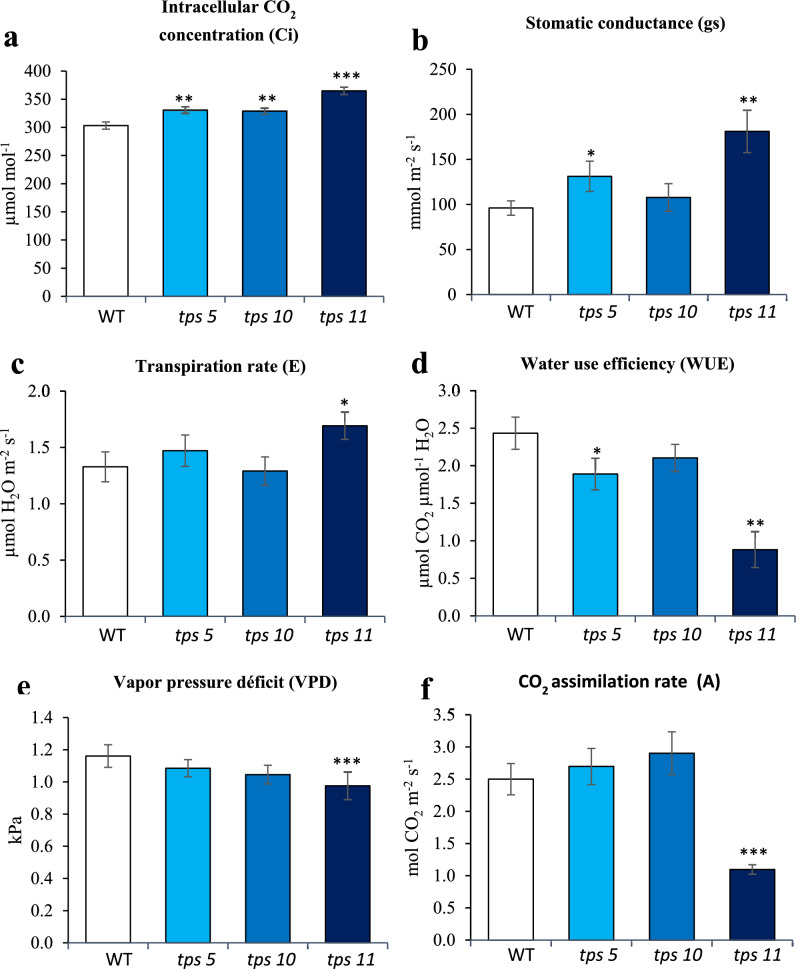
Changes in photosynthesis-related parameters in *A. thaliana tpsII* gene mutants. Intracellular CO_2_ concentration (**a**), stomatic conductance (**b**), transpiration rate (**c)**, water-use-efficiency (**d**), vapor pressure deficit (**e**), and CO_2_ assimilation rates (**f**) were measured in the *tps5-1*, *tps10-2* y *tps11-2* insertional mutants and in WT controls. The bars represent the average ± SE (*n* = 50). Asterisks over the bars denote significant differences (one-way ANOVA and Tukey test, *P* ≤ 0.05 [*], *P* ≤ 0.01 [**] or *P* ≤ 0.001 [***]). The experiment was repeated at least twice using 50 plants per genotype in each replica, all of which yielded similar results

A clear difference was observed between the photosynthetic parameters recorded in *tps5-1* and *tps10-2* mutants with those recorded for the *tps11-2* mutant. All mutants had significantly higher intracellular CO_2_ concentrations (Ci) than WT plants (Fig. 8a). Except for the significant increase and decrease in stomatal conductance (gs) (Fig. 8b) and water-use efficiency (WUE; Fig. 8d), respectively, registered in the *tps5-1* mutant, no other differences between the WT controls and the *tps5-1* and *tps10-2* mutants were observed. In contrast, the *tps11-2* mutants not only showed significantly higher *gs* and total transpiration rates (E) (Fig. 8c) but, also significantly lower WUE, vapor pressure deficit (VPD) and CO_2_ assimilation rate (A) parameters (Figs. 8e and 8f).

## Discussion

The present study provided evidence that TPS5, TPS10 and TPS11 participate in the regulation of C and N metabolism in *A. thaliana* in the context of short- and long-day photoperiods and probably in conjunction with Tre6P. They also showed that numerous parameters measured in this study, i.e., rosette size and flowering time, starch and Suc levels at the end of the short- and long-day photoperiod nights, the levels of most amino acids at the end of the short-day night, and some, such as Asn and Gln, at the end of both photoperiods´ nights, NR activity and photosynthetic parameters, in general, were significantly different in *tps11-2* mutant. This behavior is consistent with the unclear phylogenetic relationship of AtTPS11 and its orthologs, which has led some studies to place them in a clade of their own, separate from all other class II TPS proteins (Fichtner and Lunn 2021).

The first hint that C and N metabolism might be enhanced by mutations in these class II *TPS* genes was the increased vegetative growth rate and the retarded flowering of mutants, which was particularly obvious in the *tps10-2* mutant. The increased vegetative growth registered in the *tpsII* mutants could have been associated with their higher intracellular CO_2_ concentrations. On the other hand, other photosynthetic parameters, such as the significantly lower WUE and CO_2_ assimilation rate (A) detected in the *tps11-2* mutant, which had the smallest rosette area of the three *tpsII* mutants tested in this study, were contrary to the promotion of growth, except for vapor pressure deficit (VPD), which was within optimum range of values associated with plant vegetative and reproductive growth (Zhang et al. 2017; Jiao et al. 2019; Zhong et al. 2023). Therefore, yet to determined mechanisms were probably responsible for sustaining the faster growth rate observed in the *tpsII* mutant plants. This proposal is supported by numerous free air CO_2_ elevation studies that have shown that higher rates of photosynthesis do not necessarily lead to a corresponding increase in biomass and yield (Nösberger et al. 2006).

Curiously, retarded flowering in these *tpsII* mutants was inversely proportional to increased rosette diameter. In this respect, a report by Wahl et al. (2013) provided robust evidence that Tre6P was, indeed, required for the appropriate activation of flowering, through its influence on the proper expression of the day-length sensitive *FLOWERING LOCUS T* leaf gene, responsible for the long-distance signaling needed to activate flowering programs in the shoot apical meristems, and on Tre6P accumulation in leaves, needed for the regulation of signaling pathways linking plant age and existing carbohydrate reserves to flowering. Moreover, several studies have reported that oscillations in Tre6P levels in *A. thaliana*, produced by diverse mechanisms, led to precocious or delayed flowering (Schluepmann et al. 2003; van Dijken et al. 2004; Gómez et al. 2010; Wahl et al. 2013).

Based on the above evidence, the behavior of the three *tpsII* mutants analyzed in this study, could be interpreted in several, and sometimes contradictory, ways. On one hand, the delayed flowering times previously reported in the *tps1* mutant and in at least two *tpsII* mutants included in the present study suggests that at least some TPSII proteins interact positively with TPS1/Tre6P to regulate flowering. On the other hand, the significantly higher rosette diameters produced by the same mutants may be indicative that they act as negative regulators of Tre6P´s control of vegetative growth. The probable mechanism(s) involved still need to be determined but may be probably associated with the observation that the stabilization of AtTPS1 and concomitant foliar accumulation of Tre6P, was crucial for the adequate systemic transport of FLOWERING LOCUS T (Fichtner et al. 2020). Other possible mechanisms could be related with the recent findings linking the overexpression of *TPS1*, *TPS3* and *TPS5* with an accelerated flowering in *Lilium* × *formolongi* through the control of miR156 expression and the modification of the carbohydrate levels of the plant, mostly Suc (Zhang et al. 2022).

Among the NSCs examined, only starch was found to significantly increase its foliar contents in the *tpsII* mutants examined. The effect was more pronounced during the day and at the end of the 8 h night period of the two photoperiods examined. Curiously, increased starch levels were reported previously in a *tps1* mutant having plastids that accumulated large starch granules that persisted until the end of seed development, whereas a transcriptomic study of the torpedo, bent cotyledon and mature embryos of the same *tps1* mutant revealed a coordinated downregulation of genes involved in starch and sucrose degradation (Gómez et al. 2006). On the contrary, the increased starch phenotype observed in the present study did not agree with a number of studies that reported divergent starch accumulation patterns in *tpsII* mutants, such as the observation that aphid infestation in *A. thaliana tps11* mutants was probably facilitated by an augmented allocation of carbon to Suc, the main carbon and energy source for this insect pest, in detriment to starch. This proposition was further supported by the increased starch content observed in *TSP11* overexpressing plants (Singh et al. 2011). Subsequent studies similarly reported lower sucrose and higher starch contents, respectively, in transgenic cold-tolerant *A. thaliana* plants overexpressing a *TaTPS11* gene from a winter-hardy wheat (Liu et al. 2019), whereas a *tps8* mutant in *B. napus* was found to increase the carbon flow to the TCA pathway, in detriment to starch and soluble sugar contents, the former of which was more than 50% lower than in WT plants (Yuan et al. 2024b). Possible explanations for this lack of coincidence with other class II *TPS* mutants, particularly *tps11*, could be attributed to particular experimental factors such as the influence of an insect-derived signal in the carbon allocation change observed in the *tps11* mutant or the likelihood that the wheat *TaTPS11* and *B. napus TPS8* genes might have different physiological roles than those of *AtTPS11*. In this respect, a previous report indicated that *OsTPS8* could be involved in the regulation of suberin deposition in rice through ABA signaling, an effect that was suggested to contribute to the observed salt stress tolerance observed in *OsTPS8* OE plants (Vishal et al. 2019). Nevertheless, the increase in starch levels detected in the three *tpsII* mutants tested and, previously, in the *tps1* mutants described above, reinforces the possibility that these particular class II TPS proteins may somehow interact with AhTPS1-Tre6P central control point to negatively regulate starch levels in plants. This proposal is supported by the expression pattern of *TPSII* genes reported in maize, which were found to reach their maximum levels during the night, in coincidence with gradually declining starch contents (Henry et al. 2014). Other scenarios by means of which class II TPS proteins could negatively affect starch content in plants could involve an interference with the Tre6P synthase-dependent increase in redox activation of the AGPase, the control point of starch biosynthesis (Kolbe et al. 2005; Lunn et al. 2006), the phosphorylation-dephosphorylation cycle required for efficient starch breakdown and/or β-amylase activity (Martins et al. 2013).

The significantly larger rosette diameters produced in the *tpsII* mutants herewith analyzed could be also interpreted as resulting from their inability to block the decrease in sucrose levels, similar to the effect produced by the augmented Tre6P levels generated in *TPS1* OE *A. thaliana* plants (Figueroa et al. 2016). Thus, the present results reinforce the inverse relationship proposed to occur between AhTPS1 activity and these particular class II TPS proteins, and further support their possible role in the negative regulation of AhTPS1 activity, subsequent Tre6P synthesis and the control of C allocation in *A. thaliana*. This proposal is supported by the recent finding that both class I and Class II *TPS* gene isoforms were co-expressed in grapevines during various development stages and in response to exogenous Suc addition or stress conditions, leading to the suggestion that class II TPS proteins could establish a direct or indirect interaction with Tre6P biosynthetic genes (Morabito et al. 2021). In addition, a previous study in rice demonstrated that different isoforms of OsTPS1 interacted with OsTPS8 and OsTPS5 proteins, thereby suggesting their potential to modify Tre6P levels and plant development, through modified carbon allocation patterns (Zang et al. 2011).

Additionally, the behavior of these *tpsII* mutants was similar in several respects to the pattern of organic and amino acids distribution observed in *TPS1* OE *A. thaliana* plants reported by Figueroa et al. (2016). Thus, similar to the highest levels of citrate and fumarate detected in the *TPS1* OE plants at the end of the day, all *tpsII* mutants analyzed in this study predominantly reached highest citrate levels at the end of both the short and long days and generally accumulated significantly higher amounts of succinic and malic acids through the duration of the luminous short- and long-day photoperiods, as well. Likewise, fumaric acid reached levels that were higher than WT during the entire short-day photoperiod. Although most TCA pathway-related organic acids in the *tpsII* mutants remained higher than WT during the dark, sharp decreases after the 8 h phase were observed for citric and malic (short-day) and fumaric acids (long-day), but only in the *tps10-2* mutant. The sharp decrease in malic acid content observed in all plants examined during the night periods could be representative of the known transport of this organic acid to the mitochondria to sustain the respiratory TCA pathway and ATP production via oxidative phosphorylation (Lee et al. 2021). In contrast, the generalized reduction of citrate observed in the night periods could be representative of its use to sustain the TCA pathway and/or to support nitrogen assimilation via its participation in the production of glutamate or glutamine (Figueroa et al. 2016). This possibility is supported by the increased levels of both amino acids, mostly glutamine, detected in all three *tpsII* mutants. The latter could also be explained by the extensively induced NR activity levels that were detected in the *tpsII* mutants through the duration of both photoperiods examined.

Increased direction of assimilated carbon into pyruvate, not detected in the *tpsII* mutants in the present study, was assumed by the generally higher levels of Asp and Asn, Ile, Thr, Ala, Leu and Val amino acids which were found to be notably dependent on the photoperiod employed. This pattern coincided with the results reported by Figueroa et al. (2016) who found that the content of similar amino acids, such as Ala, Asp, Val, Asn, Thr and Ile, was rapidly increased in response to the increased Tre6P levels generated in transgenic *A. thaliana* plants. Also, in accordance to the data reported by Figueroa et al. (2016), Ser showed a tendency to decrease under both short- and long-day conditions, following a pattern that was the inverse of Gly. Glyceric acid levels were also higher than WT mostly during the luminous stages of the photoperiods, a pattern suggesting that these *tpsII* mutants were more prone to photorespiration than WT. However, the photosynthesis data showed that only the *tps11* mutant had an assimilation/ respiration ratio that was significantly lower than that of WT. On the other hand, the possible photorespiratory release of NH_4_^+^ by the glycine decarboxylase complex could also explain the generalized activation of NR activity and the increased Gln accumulation observed in all three *tpsII* mutants. Also, increased NR activity could have resulted from the Tre6P-mediated activation of the protein phosphatase known to catalyze the dephosphorylation of the Ser534 residue of NR (Figueroa et al. 2016).

The results obtained from the analysis of these *tpsII* mutants, in addition to published data, support a working model that contemplates the interaction of class II TPS proteins with SnRK1, a central metabolic regulator, leading to the phosphorylation of the latter and possible downregulation of the former, in order to optimize the plant´s response to metabolic stress (Glinski and Weckwerth 2005; Harthill et al. 2006; Radchuk et al. 2010; Van Leene et al. 2022). This response could involve a drastic modification in carbon partitioning, leading to altered levels of sugars and most organic and amino acids, either via the direct binding of phosphorylated TPS II proteins to Tre6P and other related molecules through their conserved ligand-binding site residues in their glucosyltransferase-like and TPP-like domains (Lunn 2007), the synthesis of a disaccharide other than Tre (Marin et al. 1998; Pade et al. 2015), and/or through development-dependent changes in the expression of *TPSI* genes/ TPS1 enzyme activity, possibly through their direct interaction with phosphorylated TPSII proteins (Lunn 2007; Zang et al. 2011).

## Conclusions

Insertional mutants of the *TPS5*, *TPS10* and *TPS11* had a significant effect on the regulation of the NSC carbon metabolism and organic acid and nitrogen metabolism, the latter measured by amino acid level fluctuations, in *A. thaliana* during both short- and long-day photoperiods. The observed effect was sometimes dependent on the type and stage of the photoperiod employed. The combined behavior of these mutants suggested that that these particular *TPSII* genes may have a negative role in the regulation of C allocation and N assimilation by the TPS1-Tre6P system in *A. thaliana* plants. Although possible mechanism(s) by means of which these TPS II proteins may specifically target TPSI activity and concomitant Tre6P levels to regulate C and N metabolism in plants in response to varying photoperiods may be inferred from the results obtained in this study, the actual biochemical process(es) remain(s) to be determined.

## Supplementary Information

Below is the link to the electronic supplementary material.

Supplementary file1 (DOCX 149 KB)

Supplementary file2 (DOCX 17 KB)

## Data Availability

The datasets generated during and/or analyzed during the current study are available from the corresponding author on reasonable request.

## Abbreviations

Glc: Glucose
NR: Nitrate reductase
Suc: Sucrose
SnRK1: Sucrose-non-fermenting1-related kinase1
Tre6P: Trehalose-6-phosphate
TPS: Trehalose phosphate synthase
TCA: Tricarboxylic acid
WUE: Water use efficiency
