# Supplementary figures and images for: The tps5, tps10 and tps11 class II trehalose phosphate synthase mutants alter carbon allocation to starch and organic and amino acids at two different photoperiods in Arabidopsis
Source: Planta. 2025 May 2;261(6):122. doi: 10.1007/s00425-025-04705-1 (PMC12048469; doi:10.1007/s00425-025-04705-1)

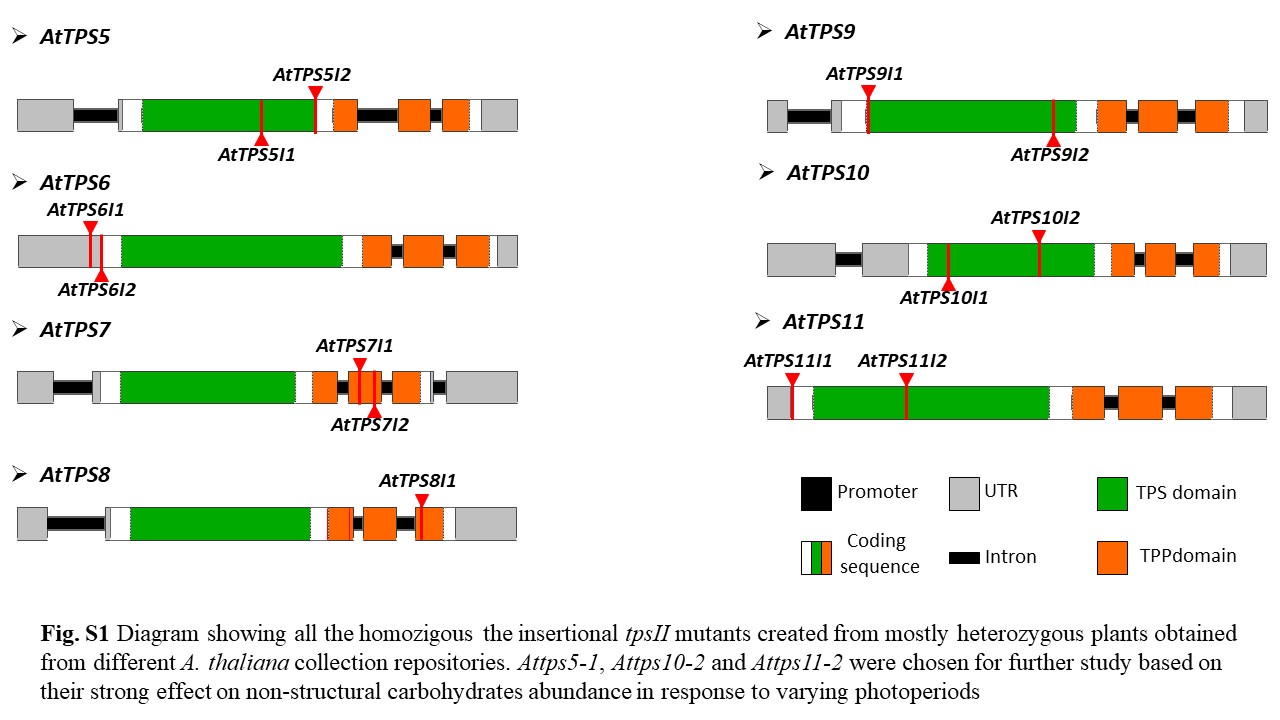

Supplement: Supplementary file 1 — Supplementary file1 (DOCX 149 KB) [file 425_2025_4705_MOESM1_ESM.docx]
