# Supplementary material for: The tps5, tps10 and tps11 class II trehalose phosphate synthase mutants alter carbon allocation to starch and organic and amino acids at two different photoperiods in Arabidopsis
Source: Planta. 2025 May 2;261(6):122. doi: 10.1007/s00425-025-04705-1 (PMC12048469; doi:10.1007/s00425-025-04705-1)
Supplement: Supplementary file 2 — Supplementary file2 (DOCX 17 KB) [file 425_2025_4705_MOESM2_ESM.docx]

**Table S1** Oligonucleotides employed for genotyping the three class II *TPS* gene mutants used in this study.

| **Gene** | **Sequence (5’→3’)** | MT (^o^C) | Direction^a^ | LP+RB (bp) | LB+RP (bp) | Polymorphism (Genotype) | Gene/Insertion |
| --- | --- | --- | --- | --- | --- | --- | --- |
| *AtTPS5*-I1-LP | CTGCGAGAGAGAGCCTAAAAAG | 63.40 | D | 1203 | 788 | SALK_007952 | *AtTPS5* |
| *AtTPS5-*I1-RP | TTTAATTTGGACTGTCCAATTTC | 60.60 | R |  |  |  |  |
| *AtTPS5*-I2-LP | AAACCTGATACCAAACTCGACG | 63.90 | D | 1151 | 473 or 817^b^ | WiscDsLoxHs015_11G |  |
| *AtTPS5*-I2-RP | AACCCGTGGTTCTGATTGATAC | 63.70 | R |  |  |  |  |
| *AtTPS5*-I2-LP-bis | TCCTTCTGTCTTTCCCACTGAC | 64.40 | D | 1161 | 944 or 1288^b^ |  |  |
| *AtTPS5*-I2-RP-bis | ACGCTGATTTGATTGGCTTTCA | 66.70 | R |  |  |  |  |
| *AtTPS10*-I1-LP | CCCAGAATATCCCATCTCTCTG | 63.60 | D | 1208 | 713 | SALK_029104.35.50.x | *AtTPS10* |
| *AtTPS10*-I1-RP | TTTTATGCACATCTTGTGGGAG | 63.60 | R |  |  |  |  |
| *AtTPS10*-I2-LP | CCTTTACAATCGAGGTCTCAGG | 63.70 | D | 1168 | 782 | SALK_110873.18.25.x |  |
| *AtTPS10*-I2-RP | AGGGTTAAGCTTGGTTTCTTCC | 63.30 | R |  |  |  |  |
| *AtTPS10*-I3-LP | TGATCAGGTCACCTTCTCTGTG | 64.30 | D | 1133 | 622 | GK-304B04-015592 |  |
| *AtTPS10*-I3-RP | CAATCCAAGCATTCTACAGCAG | 63.60 | R |  |  |  |  |
| *AtTPS11*-I2-LP | AGACCAGCTTAGTCTGGTTTCG | 63.50 | D | 1042 | 406 or 750^b^ | WiscDsLox293-296invP2 | *AtTPS11* |
| *AtTPS11*-I2-RP | GTTCTGTTCAAGAAGCTGACCC | 64.00 | R |  |  |  |  |
| *AtTPS11*-I1-LP | AAGCCCACAAAGTCTCAGAAAC | 63.40 | D | 1051 | 569 | GK-592G12-021782 | Intergenic |
| *AtTPS11*-I1-RP | GAATCGTGGTCCATACAAAATG | 62.80 | R |  |  |  |  |

a: With respect to the direction of the insertion.

b: Depending on the WISC oligonucleotide used for amplification.
